# Clinical, Histopathological and Immunohistochemical Aspects of Digital Papillary Adenocarcinoma: A Case Report and Literature Review

**DOI:** 10.5146/tjpath.2023.01606

**Published:** 2024-01-22

**Authors:** Sergen Yagcı, Aysen Terzi, Abbas Albayati, Ahmet Cagrı Uysal

**Affiliations:** Department of Pathology, Başkent University Faculty of Medicine, Ankara, Turkey; Department of Plastic and Reconstructive Surgery, Başkent University Faculty of Medicine, Ankara, Turkey

**Keywords:** Malignant eccrine tumor, Digital papillary adenocarcinoma, Mohs Micrographic Surgery

## Abstract

Digital papillary adenocarcinoma (DPA) is a rare malignant eccrine tumor. A 62-year-old female presented with a subcutaneous nodular 1.5cm-mass in the thumb. Macroscopically, a poorly circumscribed mass containing cystic and solid components was observed. Microscopically, epithelial neoplasm consisting of tubular-cystic structures with back-to-back arrangements was observed. The lining epithelium was composed of cuboidal/columnar cells with mild atypia, with micropapillary extensions. Immunohistochemistry revealed double-layered neoplastic epithelium containing two different types of cells: basaloid/myoepithelial and luminal. We recommend two out of vimentin, HMWCK, and D2-40 for myoepithelial/basaloid cells, also CK7 and EMA for luminal/columnar cells. As the tumor had infiltrated the surgical margins, the patient underwent axillary sentinel lymph node (SLN) dissection and re-excision with Mohs micrographic surgery (MMS). Two additional MMS stages were required due to suspicious surgical margin positivity in the frozen sections. The operation was continued despite the risk of loss of function. Upon examination of the permanent sections, we observed no tumors in the suspected positive foci. Additionally, no tumor was found in the surgical margins. No metastasis was detected in the sentinel lymph node. We have reached 300 reported cases of DPA in the literature. We discussed the histopathological and intraoperative diagnostic pitfalls of DPA with a literature review and our experience.

## INTRODUCTION

Digital papillary adenocarcinoma (DPA) is a rare malignant tumor of the sweat glands that often presents as a solitary painless mass on the digits or toes with an incidence of 0.08 per 1 000 000 people/year (1) and was originally described by Helwig (2) in 1984. The male/female ratio was reported 4:1 (1). It is most frequently diagnosed in the sixth to eighth decades but adolescents may also be affected (3). Most tumors exhibit nodular and cystic growth patterns with a median diameter of 1.7 cm. Due to its slow growth and non-specific symptoms and signs, the diagnosis is often missed or delayed. The tumor has a frequently inconspicuous clinical course but significant potential for recurrence and metastasis. The overall local recurrence rate has been reported as 30%. However, after adequate surgical treatment with re-excision or amputation, this rate may be decreased to 5%. The rate of distant metastases has been reported to be 14-26% regardless of treatment or the presence of local recurrence (4,5). The recommended treatment is surgical with clear resection of the margins. There is a possibility of proximal amputation in case of muscle and bone invasion (4,5). Because of the difficulty of its differential diagnosis from metastatic adenocarcinoma, this process is problematic for the pathologist (6). Here, we aimed to discuss the case of DPA with the diagnostic pitfalls and review the literature.

## CASE REPORT

**Figure 1:**
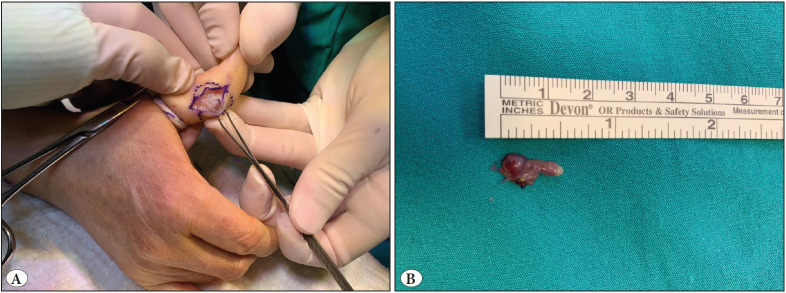
A subcutaneous nodular 1.5cm-mass in the thumb. No ulceration or color change (**A and B**).

**Figure 2:**
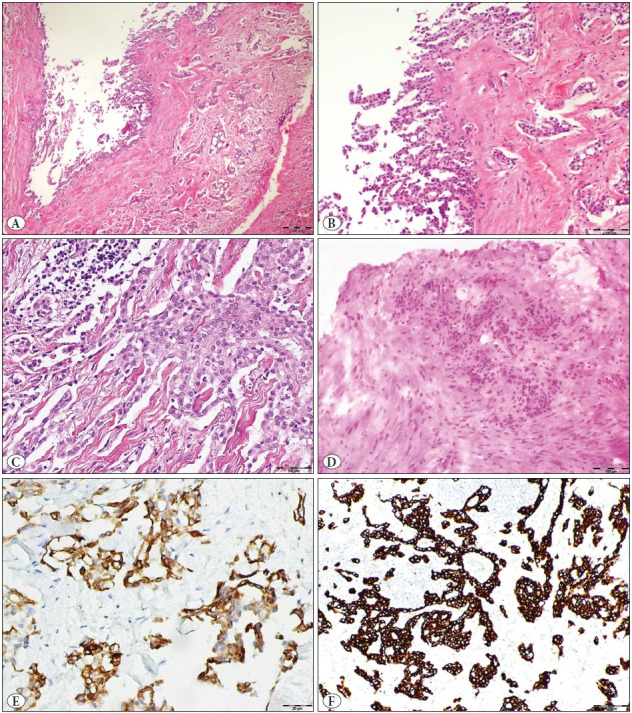
**A**) An epithelial neoplasm was observed in the solid component, some of which consisted of cribriform, back-to-back tubular structures (hematoxylin and eosin x50). **B**) The lining epithelium of the cystic component was composed of a single row of flattened mild atypical cells; sometimes it contained double-layered cuboidal/columnar cells with mild atypia, with micropapillary extensions to the lumen (hematoxylin and eosin x100). **C**) Epithelial cells with mild atypia form solid sheeting, focally (hematoxylin and eosin x200). **D**) Suspicious positivity was noted in frozen sections. It was noticed that there was no tumor in the suspicious positive foci in the permanent sections (hematoxylin and eosin x200). Immunohistochemistry revealed that the bilayer neoplastic epithelium contained two distinct cell types; basaloid/myoepithelial and luminal. Luminal cells were CK7 positive (**E, x200**); basaloid/myoepithelial cells were HMWCK positive (**F, x100**).

A 62-year-old female presented with a painless swelling in the thumb. Clinically, it was thought to be a ganglion cyst or vascular lesion on initial physical examination. A subcutaneous nodular mass with a diameter of 1.5 cm was detected in the proximal phalanx (Figure 1). Magnetic resonance imaging (MRI) showed a cystic lesion, thought to be compatible with infectious processes, and the surgeon excised the lesion. Macroscopically, a partially well-circumscribed but mostly infiltrative mass containing cystic and solid components was observed (Figure 1). Microscopically, an epithelial neoplasm was observed in the solid component, some of which consisted of cribriform, back-to-back tubular structures (Figure 2). The lining epithelium of the cystic component was composed of a single row of flattened mild atypical cells, sometimes containing double-layered cuboidal/columnar cells with mild atypia, with micropapillary extensions to the lumen (Figure 2). A rare mitotic figure was seen. No necrosis, lymphovascular or perineural invasion was observed. Histochemical staining showed focal intracytoplasmic mucin in neoplastic cells.

Immunohistochemistry revealed that the bilayer neoplastic epithelium contained two distinct cell types: basaloid/myoepithelial and luminal (Figure 2). Tumor cells were cytokeratin AE1/AE3, MOC31, BEREP4 positive, but S100 and CEA were negative. Luminal cells were CK7 and EMA positive; basaloid/myoepithelial cells were vimentin, p63, HMWCK, D2-40, and calponin positive. We observed sparse mitotic figures and calculated the Ki-67 index as 15% in the highest areas. No atypical mitosis or necrosis was observed. The tumor was infiltrating the surrounding adipose tissue and was present in the deep surgical margins. We reported histomorphological and immunohistochemical findings consistent with DPA. We suggested re-excision if metastatic adenocarcinoma was excluded clinically and radiologically. No involvement was found in any other focus on PET/CT. Re-excision was performed with axillary sentinel lymph node (SLN) dissection and MMS. We did not detect SLN metastases. During the MMS procedure for re-excision, intraoperative examination revealed suspicious tumor foci at the surgical margins by the pathologist. Two additional MMS steps were then performed to ensure negative surgical margins. We noticed that there was no tumor in the suspected positive foci in the permanent sections (Figure 2). There was no tumor in the surgical margins. Our patient was recurrence-free after 30 months of postoperative follow-up.

## DISCUSSION

Kao et al. first defined the cases as adenoma and adenocarcinoma in their 57 cases of DPA, in 1987 (7). Duke et al. described 67 cases (50 of adenoma, 17 of adenocarcinoma), 6 cases with metastases, and three of these cases were found to have histopathological criteria previously defined as adenoma by Kao and colleagues. The results showed that only DPA terminology should be used (4).

We have found that 300 cases have been reported in the PubMed using the search terms ‘aggressive DPA’, ‘DPA’, and ‘aggressive digital papillary adenoma’ as of April 2023. The male/female ratio was 3.86 (174:45). The median age was 51 years (range, 14-96). The tumor was mostly localized on the hand, especially on the fingers (mostly third finger). On the feet, it was mostly localized on the big toe. Localization was mostly on the volar surface of the hands or feet and distal fingers or toes.

DPA is a diagnostic challenging tumor, and treatment is often delayed due to misdiagnosis (8). Metastatic adenocarcinoma and benign adnexal tumors are the two main entities that cause difficulties in the histopathological differential diagnosis. Typical histopathological features of DPA are multinodular, solid, and cystic development, but pure solid cases have also been reported (4). In addition to papillary projections protruding into the cystic lumen, tubular structures surrounded by an outer neoplastic myoepithelial layer and an inner low columnar/cuboid epithelium, with back-to-back arrangements were also observed, which are typical features of DPA. Suchak et al. suggested that the presence of tumor-associated myoepithelial cells should not be interpreted as benign but rather a clinical or histopathological evaluation for the primary adnexal origin of the tumor (5). DPA is defined as a poorly circumscribed tumor involving the dermis and subcutis (4). Since metastatic adenocarcinoma is the first line in the histopathological differential diagnosis, immunohistochemical revealing of the different phenotypes of the myoepithelial layer and columnar epithelium was valuable in our case.

Cases presented in the literature have been evaluated with a wide variety of immunohistochemical panels, and there is no recommendation for an optimal diagnostic panel for DPA. Therefore, we also reviewed the immunohistochemical markers used for differential diagnosis of DPA in the literature and showed that most of them in Table 1. We created a mini-panel recommendation for using the DPA diagnosis. We found it helpful to apply two of three markers for myoepithelial/basaloid cells vimentin, HMWCK, and D2-40. In our experience, other myoepithelial cell markers such as S100 did not stain any neoplastic cells, and p63 stained myoepithelial/basaloid cells selectively but not all of them. The most useful luminal/columnar cell markers were CK7 and EMA. MOC31 and BerEP4 are positive in most carcinomas; both were positive in our case and not helpful to distinguish DPA from metastatic adenocarcinomas. The p53 nuclear positivity of the tumor was less than 10%, which helped us to lower the probability of metastatic carcinoma. Since p53 can also show diffuse positivity in benign adnexal tumors, it did not help us in the differential diagnosis of DPA from benign skin adnexal tumors. However, the Ki-67 proliferation index can be a useful marker, as it may indicate a significant focal increase in DPA. Wide excision or digital amputation with or without SLND followed by close, long-term follow-up is the recommended treatment method of DPA (5). Six cases of Mohs micrographic surgery (MMS) have been reported in the current literature. (4,8–11). MMS offers the advantage of achieving histologic margin clearance and functional preservation (9). In our experience, two additional MMS stages were required due to suspicious positivity, unlike the previously reported 6 DPA cases that underwent MMS. Although the diagnosis is known preoperatively, suspicious positivity was noted in frozen sections because of the innocent histomorphology of DPA. The operation was advanced despite the risk of loss of function. In contrast, the residual tumor was only in a microscopic focus in the permanent sections. It was noticed that there was no tumor in the suspicious positive foci in the permanent sections. Additionally, no metastasis was detected in serial sections in the sentinel lymph node, which showed a 3.5 cm fatty change, in our case.

**Table 1:** Immunohistochemistry was applied to 111 cases, including our case*. The statistics of the immunohistochemical analysis applied in the literature are shown in the table.

**Immunohistochemistry**	**Percentage of positivity**	**Number of cases tested**
HMWCK	100%	3*
Vimentin	100%	3*
CK7	100%	29*
D2/40	100%	7*
CK AE1/AE3	100%	17*
p53	100%	7*
BerEP4	100%	2*
MOC31	100%	1*
p40	100%	8
p63	95.2%	42*
EMA	92.8%	42*
CK77	85.7%	14
Calponin	80%	10*
SMA	79.1%	48*
S100	78%	51*
CEA	68.7%	48*
p16	50%	8
ER	44.4%	9
CK 5/6	67.7%	3
Ki-67	Mean 14.9%	16*
Desmin	0%	2

Sentinel lymph node procedure was applied to 48 cases reported, and metastasis was observed in 6 cases (13%) (4,12–14). Disease-related death was reported in 6 cases (3.3%) (4,14–18), 37 patients (19.5%) showed local recurrence, and 26 patients (13.7%) had distant metastasis in the reported 190 patients with follow-up, in the current literature. According to these data, the event-free survival rate was 76.8% during the mean 57 months of follow-up. Our patient was recurrence-free after 30 months of postoperative follow-up.

DPA has a silent clinical course and innocent histomorphology. Careful histopathological examination and clinical correlation are essential in the differential diagnosis, since there are various diagnoses from benign skin-appendix tumors to metastatic adenocarcinoma. These tumors are often unrecognized because of their rarity so being aware of this entity is essential for both pathologists and clinicians.

## Conflict of Interest

The authors declare that they have no conflicts of interest.
